# Effects of repeated exposure to ozonated water on hair fibre structure and integrity: Part I

**DOI:** 10.1111/ics.70099

**Published:** 2026-05-13

**Authors:** Pedro Augusto Laurindo Igreja Marrafa, Shamish Ganpule, Vanessa Castro‐Haly, Adriana Barrinha Fernandes, Ernesta Malinauskyte, Carlos Jose de Lima

**Affiliations:** ^1^ Instituto de Engenharia Biomédica Universidade Anhembi Morumbi São José dos Campos São Paulo Brazil; ^2^ TRI Princeton Princeton New Jersey USA; ^3^ Centro de Inovação, Tecnologia e Educação (CITÉ) São José dos Campos São Paulo Brazil

**Keywords:** consumer‐relevant testing, microscopy, ozone hair treatment, spectroscopy, tensile testing

## Abstract

**Objective:**

Ozone has recently been marketed as a hair‐revitalizing treatment claimed to enhance shine, softness and overall fibre quality. However, given its strongly oxidative nature, rigorous evaluation is required to determine its true effects on hair structure. This study investigates the impact of repeated exposure to ozonated water on a variety of hair‐fibre properties.

**Methods:**

Multiple analytical techniques were used: Fourier transform infrared spectroscopy (FTIR) to detect chemical changes; scanning electron microscopy (SEM) and fluorescence microscopy to assess surface and internal morphological alterations; differential scanning calorimetry (DSC) to analyse keratin and matrix thermal behaviour; tensile testing to determine mechanical responses; and combability, shine and colour measurements to evaluate consumer‐relevant attributes.

**Results:**

Ozonated water induced distinct oxidative modifications across structural, chemical and functional domains. FTIR revealed progressive increases in cysteic acid–related bands and carbonyl groups, confirming protein and lipid oxidation. SEM and fluorescence imaging showed cuticle fusion and increased cortical fluorescence, consistent with elevated carbonyl content. Morphological cuticle changes, together with increased cysteic acid content, aligned with the rise in combing force. DSC demonstrated an increased denaturation temperature accompanied by a slight reduction in enthalpy, indicating altered crosslink density and partial disruption of keratin order; these changes were further supported by increased post‐yield stiffness in tensile tests and modifications observed in the Amide I and II regions. Changes in shine and colour were minimal.

**Conclusions:**

Prolonged exposure to ozonated water induced cumulative oxidative modifications of hair proteins, lipids and pigments, resulting in alterations to various hair properties. Ozone appears to induce a unique damage signature that extends beyond the cuticle into the cortex. Future studies will explore lipidomic and biochemical changes in greater depth and evaluate how ozone‐treated hair responds to subsequent cosmetic and environmental stressors.

## Graphical abstract

Repeated exposure to ozonated water caused cumulative oxidative changes in hair, altering structural integrity, thermal behaviour and mechanical properties. The findings reveal a distinct ozone‐induced damage profile that progresses beyond the cuticle, challenging claims of hair‐revitalizing benefits.

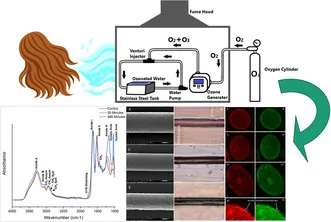

## Résumé

**Objectif:**

L'ozone a récemment été commercialisé en tant que traitement revitalisant pour les cheveux, prétendant améliorer la brillance, la douceur et la qualité globale des fibres. Cependant, étant donné sa nature fortement oxydative, une évaluation rigoureuse est. nécessaire afin de déterminer ses véritables effets sur la structure des cheveux. Cette étude examine l'impact d'une exposition répétée à l'eau ozonée sur diverses propriétés de la fibre capillaire.

**Méthodes:**

Plusieurs techniques analytiques ont été utilisées: la spectroscopie infrarouge à transformée de Fourier (IRTF) pour détecter les variations chimiques; la microscopie électronique à balayage (MEB) et la microscopie à fluorescence pour évaluer les altérations morphologiques de surface et internes; la calorimétrie différentielle à balayage (DSC) pour analyser le comportement thermique de la kératine et de la matrice; des essais de traction pour déterminer les réponses mécaniques; et des mesures de peignage, de brillance et de couleur pour évaluer les attributs pertinents pour le consommateur.

**Résultats:**

L'eau ozonée a induit des modifications oxydatives distinctes dans les domaines structurels, chimiques et fonctionnels. La spectroscopie IRTF a révélé une augmentation progressive des bandes liées à l'acide cystéique et des groupes carbonyles, confirmant l'oxydation des protéines et des lipides. La MEB et l'imagerie par fluorescence ont montré une fusion de la cuticule et une augmentation de la fluorescence corticale, compatible avec une teneur élevée en carbonyles. Les modifications morphologiques de la cuticule, associées à une augmentation de la teneur en acide cystéique, étaient corrélées à l'augmentation de la force de peignage. La DSC a montré une augmentation de la température de dénaturation accompagnée d'une légère réduction de l'enthalpie, indiquant une altération de la densité de réticulation et une perturbation partielle de l'ordre de la kératine; ces variations ont été étayées par une rigidité accrue après la limite d'élasticité lors des essais de traction et par les modifications observées dans les régions de l'amide I et II. Les variations de brillance et de couleur étaient minimes.

**Conclusions:**

Une exposition prolongée à de l'eau ozonée a induit une modification oxydative cumulée des protéines, des lipides et des pigments capillaires, ce qui a entraîné une altération de diverses propriétés capillaires. L'ozone semble induire des dommages qui ont un profil particulier, s'étendant au‐delà de la cuticule, jusque dans le cortex. De futures études exploreront plus en profondeur les variations lipidomiques et biochimiques et évalueront comment les cheveux traités à l'ozone répondent aux facteurs de stress cosmétiques et environnementaux ultérieurs.

## INTRODUCTION

Hair, though biologically classified as a skin appendage, has lost much of its evolutionary function in humans. Nevertheless, its relevance in social and emotional contexts remains significant [1]. Thus, a continuously growing personal care industry focuses on the development of products and procedures aimed at enhancing the aesthetics, care and maintenance of the hair fibre [2, 3].

Human hair has been studied in various domains beyond cosmetics. It serves as a biomarker in forensic toxicology, environmental contamination monitoring and nutritional assessments. Additionally, hair has been investigated in medical research to assess the impact of chemical agents used in hair care products and their possible correlation with disease development. It has also played a role in archaeological studies as a preserved biological specimen [4].

The structure of the hair cuticle plays a crucial role in regulating the penetration of substances into the hair shaft [5, 6]. Beneath the cuticle lies the cortex, which is the central and most substantial part of the hair fibre. It is primarily responsible for providing mechanical strength. It consists of longitudinally oriented keratin microfibrils, within which melanin granules contribute to determining the natural colour of the hair [3, 5, 6]. Human hair is primarily made up of protein, accounting for approximately 65% to 95% of its total mass. The rest is composed of water, lipids, pigments and trace elements. From a chemical standpoint, keratin, a fibrous protein, constitutes around 80% of the hair's structure [7, 8].

Alterations in hair structure generally begin at the scalp and may be attributed to either natural factors or cosmetic interventions. These modifications often result from oxidative processes triggered by ultraviolet radiation from sunlight, as well as from treatments such as bleaching, dyeing, permanent waving and hair straightening [9].

Ozone (O_3_) is a triatomic oxygen molecule known for its powerful oxidative properties. It occurs both naturally and synthetically and is widely used in industrial, environmental and medical applications [10, 11, 12]. In its gaseous form, ozone has been utilized for disinfecting, deodorizing and decontaminating various environments. Ozonated liquids have found applications in wound care, food processing and water treatment, with increasing interest in their clinical and cosmetic use [13, 14, 15, 16].

As ozone gains traction in the healthcare sector, numerous studies have evaluated its applications both as a bactericidal and fungicidal agent, as well as its potential in the treatment of wounds and other skin‐related conditions. When used for its antimicrobial properties, ozone acts by decomposing into O_2_ and a single oxygen atom (O^1^), the latter being highly reactive. This reactivity disrupts bacterial cell walls and alters the structure and function of proteins and carbohydrates. In wound treatment, ozone is often applied through ozonated water in a method known as hydrotherapy. In this form, it has been shown to ameliorate tissue fluid effusion, reduce inflammatory responses, promote wound healing and alleviate pain and pruritus. The therapeutic use of ozone has already been investigated for a variety of dermatological conditions, including the treatment of diabetic foot ulcers and interstitial cystitis [17, 18, 19, 20, 21, 22, 23].

Companies within the cosmetic and beauty industries have begun adapting this compound for use in personal care. In particular, the application of ozonated water in hair treatments has emerged as a novel trend, often marketed with claims of improved shine, softness and strength [24, 25, 26, 27]. However, these claims are largely based on anecdotal evidence or presented in brochures, with limited scientific literature supporting the safety and efficacy of ozone exposure on human hair fibres, including a published study suggesting the replacement of personal care products with ozonated water for skin and hair cleansing processes, as well as a previous study published by our group which, to the best of our knowledge, was the first to specifically investigate the oxidative damage induced by ozone on the hair fibre [28, 29, 30, 31, 32].

Murphy suggests that ozone's oxidative potential is greater than that of hydrogen peroxide [33]. Therefore, its repeated application to hair raises concerns regarding potential structural damage. Previous reports have identified geranyl acetone, 6‐methyl‐5‐hepten‐2‐one and decanal as typical carbonyl end‐products formed when ozone reacts with squalene, cholesterol and unsaturated fatty acids in tissues, serving as chemical indicators of ozone‐induced oxidative stress on the skin and other lipid‐rich surfaces [34, 35]. Moreover, ozone has been shown to deplete essential antioxidants such as vitamins C and E in the skin and to induce lipid peroxidation in the upper layers of the epidermis. These oxidative processes may compromise skin barrier function and trigger inflammatory responses, affecting not only the scalp but also potentially the emerging hair shaft [34, 36, 37].

The growing use of ozone‐generating devices in beauty salons underscores the need for rigorous, independent investigation of the effects of hair repeated exposure to ozonated water. This study evaluates virgin human hair exposed in vitro to varying cycles of ozonated water to test industry claims of cosmetic benefit and to identify potential structural damage, thereby providing an evidence‐based perspective on ozone in hair treatments.

## MATERIALS AND METHODS

Medium brown, European origin, hair tresses with approximately 20.3 cm in overall length, 2.5 cm wide and weighing about 3 g were obtained from International Hair Importers & Products, Inc. (Glendale, New York).

### Washing the samples with ozonated water

The hydrodynamic system was composed of a stainless steel tank for water (1 L volume), flexible silicone ducts, a centrifugal hydraulic pump, a Venturi injector, an ozone generator and an oxygen cylinder (Figure 1). The ozone feed rate adjusted for this protocol was 5.5 mg/min. The applied ozone dosages were calculated as a function of the exposure time and the sample area (cm^2^) as proposed by van Leeuwen [38]. The ozonated water was continuously pumped by a centrifugal‐type pump with a flow rate of 35 mL/s. The ozone generator (Allos O3, HTM Ltda, Brazil) was calibrated and adjusted to a concentration of 45 mg/L, with an oxygen gas supply rate at the inlet of 0.125 L/min.

**FIGURE 1:**
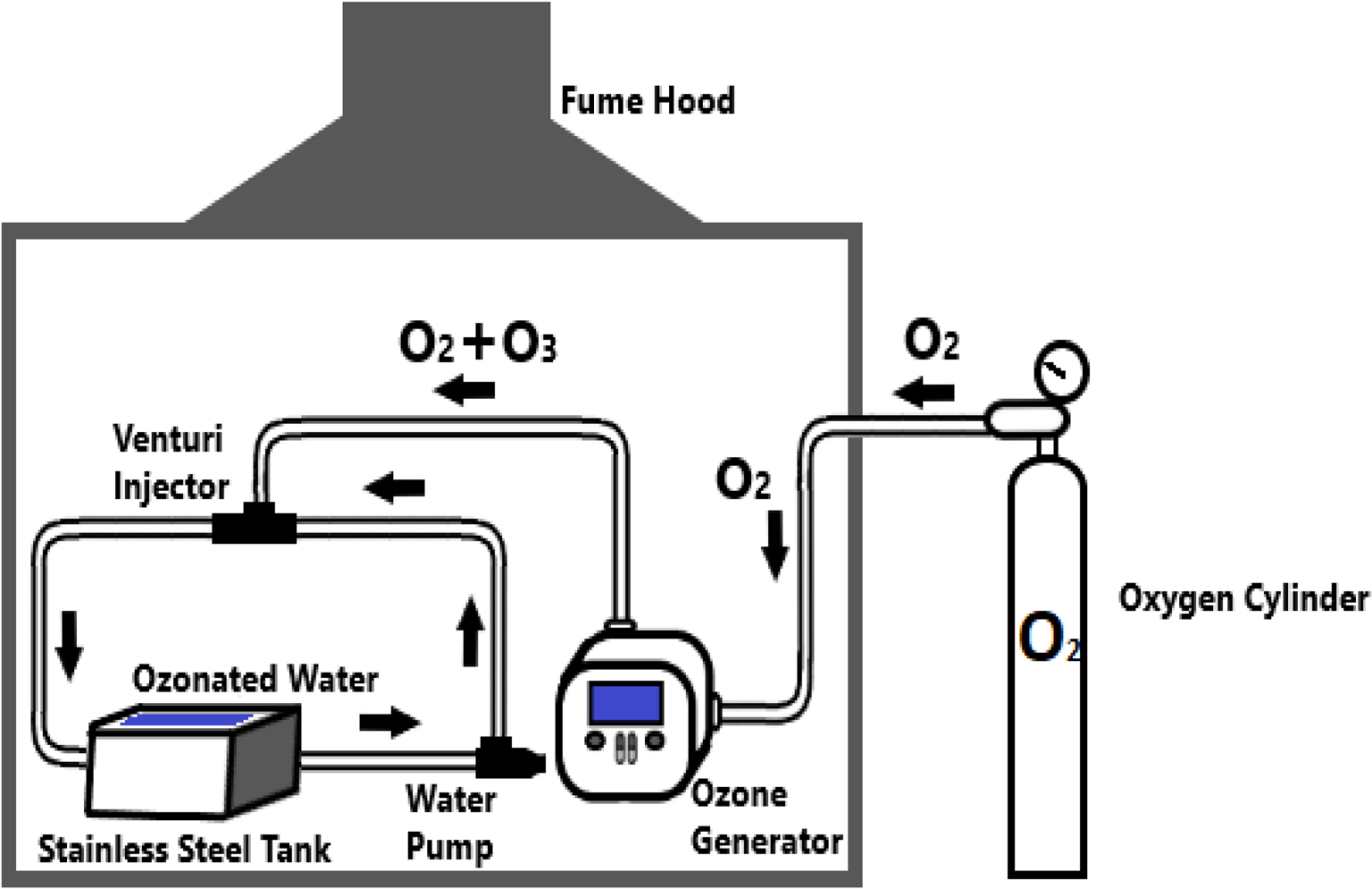
Schematic representation of the assembly for exposure of the hair fibre to the dynamic ozonated water.

### Digital microscopy

Hair surface changes were evaluated using a Keyence VHX‐X1 3D Digital Microscope system (Keyence, Japan), equipped with an automatic stage to enable a high‐resolution (4K) analysis. Images of 10 fibres per treatment group were captured at 500x magnification.

### Scanning electron microscopy (SEM)

10 fibres per hair sample were affixed to 2.5 cm diameter metal stubs using a double‐sided copper adhesive tape; subsequently, the samples were coated with 7 nm platinum using DST1 High Vacuum Sputter Coater (Vac Coat Ltd., London, UK). The micrographs were captured using a scanning electron microscope (SEM), model JSM‐IT200 (JEOL, Tokyo, Japan), operating at a beam voltage of 7 kV and a working distance of ~11 mm.

### Fluorescence microscopy

Hair fibres were embedded as a bundle in DI Water and frozen using dry ice prior to cryosectioning. Several hair cross‐sections (2 μm) were collected for image analysis. Hair cross‐section images were collected on a Eclipse TE2000‐U microscope (Nikon Corporation, Tokyo, Japan), equipped Prior Lumen 200 Fluorescence Illumination System (Prior Scientific Instruments Ltd., Cambridge, UK) and a Nikon DS‐Qi2 monochrome camera (Nikon Corporation, Tokyo, Japan). At least 10 images of different fibres per treatment group were acquired using the NIS‐Elements software (Nikon Corporation, Tokyo, Japan). Fluorescence images were acquired in the red channel (CY3 filter; Excitation: 560/40 nm; Emission: 630/75 nm), exposure 147 ms at 100X magnification, in the green channel (GFP filter; Excitation: 475/35 nm; Emission: 530/43 nm), exposure 227 ms at 100X magnification.

### Infrared spectroscopy

Attenuated total reflectance–Fourier transform infrared spectroscopy (ATR‐FTIR) spectra were recorded on Splotlight System 400 using an ATR‐FTIR accessory (PerkinElmer, USA) with Pike Miracle ATR. 10 spectra were collected at 6 cm hair length for each sample. The samples were evaluated at the 0 min, 30 min and 360 min time points. The same hair bundle was measured at all time points. All spectra were recorded from 4000 to 650 cm^−1^ accumulating 8 scans at a resolution of 1cm^−1^. The data were analysed using GRAMS/AI (Thermo Fisher Scientific, Waltham, Massachusetts, USA). Origin Pro software (v. 8.5) was used to plot the spectra.

### Single fibre tensile testing

Before testing, the hair samples were conditioned at 60 ± 2% relative humidity (RH) and 20 ± 2°C for a minimum of 24 h. The fibres were secured using brass clamps, and their dimensions were measured at six different points under the same humidity conditions using a laser scanning micrometre (FDAS770 Fibre Dimensional Analysis System, Diastron Ltd., Andover, UK). A total of fifty fibres from each group were stretched at a rate of 40 mm/min. Stress–strain profiles were obtained in a wet state using the MTT690 Miniature Tensile Tester (Diastron Ltd., Andover, UK). Mechanical properties were processed with UVWin 4.3 software (Diastron Ltd., Andover, UK), and Young's modulus was determined by evaluating the slope of the linear region of the curve (0.1–0.5% strain).

### Differential scanning calorimetry

Thermal properties of hair were tested using the DSC2500 instrument (TA Instruments, DE, USA). Six replicates were prepared for each sample. For this study, the same hair and equilibration conditions were used as for the fibre dimension study. Approximately 10 mg of finely chopped hair was placed in a high‐pressure hermetic DSC pan together with 50 μL of DI water. The heating rate was 10°C min^−1^. Prior to testing, the instrument was calibrated using an indium standard.

### Dry combing analysis

Hair friction was tested using Instron Tensiometer 3343 equipped with a 50 N load cell (Instron Corporation, Norwood, MA, USA). The hair tress is pulled through a comb, while the load cell records the resulting frictional forces. Each tress is subjected to six passes. Each group consisted of six tress replicates. To prevent cross‐contamination, the comb is replaced between each experimental group. Prior to testing, hair tresses are equilibrated overnight at 60 ± 2% RH and 20 ± 2°C temperature.

### Colour analysis

Colour variation is assessed by measuring the CIELAB L*a*b* parameters using a portable colorimeter (WF32, FRU, Shenzhen, China). Measurements are taken from six hair tresses, with ten distinct areas analysed per tress (five points on each side). The overall colour change in hair was calculated using the formula below:

$$∆E=\sqrt{{\left(∆L\right)}^{2}+{\left(∆a\right)}^{2}+{\left(∆b\right)}^{2}}$$

### Shine analysis

Hair shine was evaluated using the SAMBA Hair v3 system (Bossa Nova Technologies, USA). Six hair tresses were prepared for each experimental group and mounted onto an in‐house–developed cylindrical holder. A comb was used during mounting to ensure consistent fibre alignment. Each tress was measured at the top, middle and bottom on both sides, resulting in a total of 36 measurements per group. Shine (Reich–Robbins) was determined through the simultaneous acquisition of specular and diffuse images, followed by data processing using the SAMBA Hair® v3.03 software.

### Statistical analysis

The analysis was performed with the JMP analytical software (SAS Institute Inc., NC, USA) using parametric one‐way ANOVA and Tukey's HSD test to differentiate treatment groups (*α* = 0.05). *p*‐values less than 0.05 were considered statistically significant and are indicated in the graphs using different letters for statistically different groups. The data in the graphs are presented as means ± standard error of the mean.

## RESULTS AND DISCUSSIONS

The ozone measurement in water suggests that hair was exposed to 3.5 mg/L of ozone in water.

### Hair microscopy

Although ten images were collected for analysis, Figure 2 presents one representative SEM and one representative digital microscopy image for each group: the control (Figure 2a,b), the 30‐min ozonated‐water exposure (Figure 2c,d) and the 360‐min exposure (Figure 2e,f).

**FIGURE 2:**
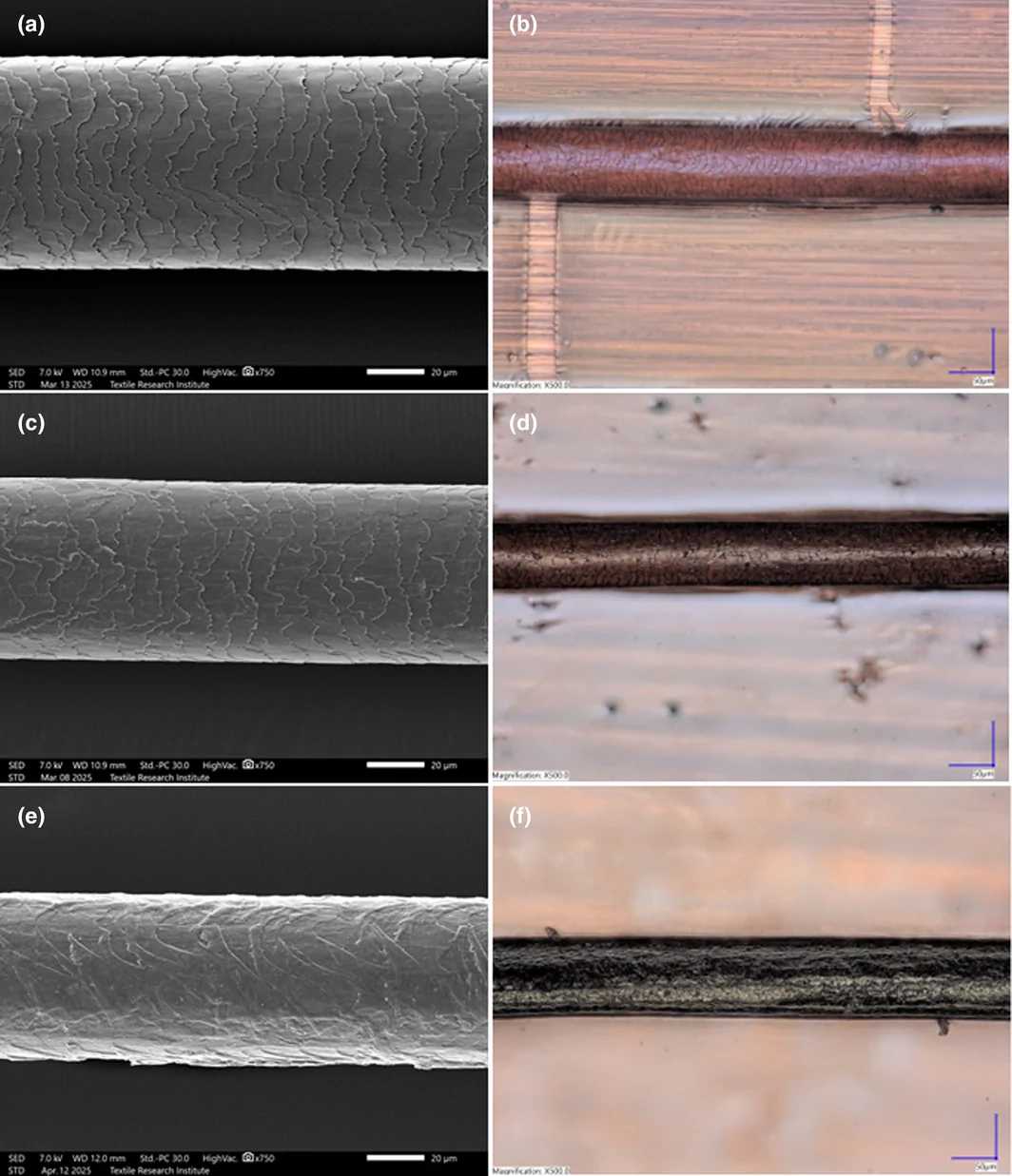
SEM microscopy images (750× magnification): (a) untreated control; (c) hair exposed to ozonated water for 30 min; (e) hair exposed to ozonated water for 12 × 30 min. Digital microscopy images (500× magnification): (b) untreated control; (d) hair exposed to ozonated water for 30 min; (f) hair exposed to ozonated water for 12 × 30 min.

The micrographs from the control and 30‐min groups showed no appreciable differences in surface morphology. By contrast, the 360‐min exposure produced marked alterations in the hair‐fibre surface, characterized by severe modification of the cuticular architecture. The step‐edges appeared smoothed and partially merged, giving the surface an almost continuous appearance.

Cuticle loss has previously been reported by Robinson [39], who observed that intense washing following bleaching resulted in loss of cuticles at the distal ends of hair fibres. However, this differs from the present findings: As shown in Figure 2, cuticle modifications in our study occurred along the entire fibre length, despite the absence of any mechanical manipulation such as massaging. The alterations observed here resemble those described for severe oxidative damage, including bleaching at 60°C for 90 min [40] and long‐term UV exposure combined with bleaching [41]. Robbins provided an in‐depth analysis of similar structural changes and proposed that UV exposure crosslinks structural boundaries into amorphous regions, after which bleaching further fuses or dissolves portions of the cuticle, explaining the reduced differentiation between cuticle layers [41].

Fluorescence microscopy was used to qualitatively assess chemical changes following hair ozonation. Ten images were captured for each fluorescent filter in each test group, and one representative image is shown in Figure 3.

**FIGURE 3:**
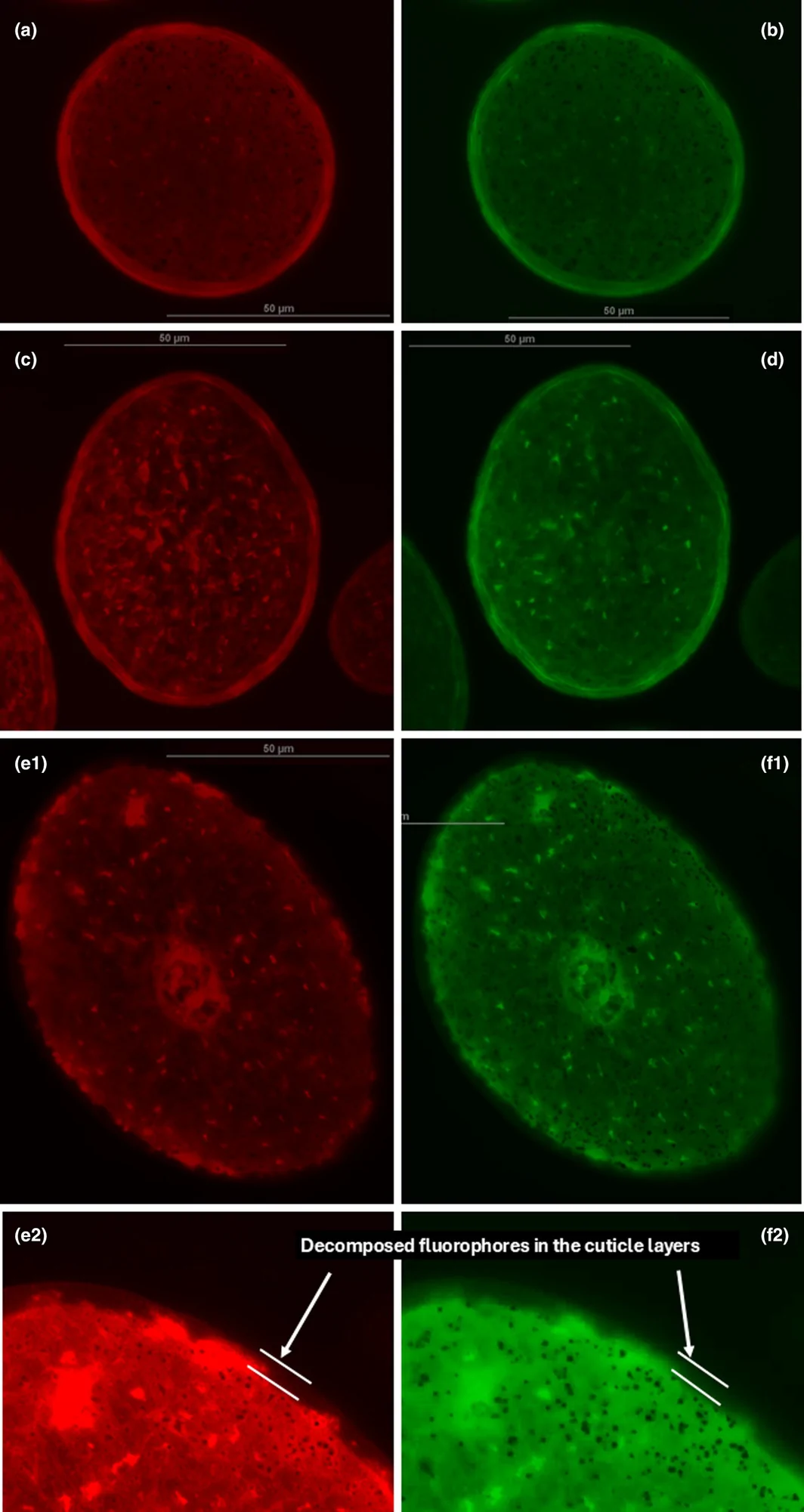
Fluorescence microscopy images (100× magnification): (a, b) untreated control; (c, d) hair exposed to ozonated water for 30 min; (e, f) hair exposed to ozonated water for 12 × 30 min. Red and green filters were applied, respectively. Subfigures labelled ‘1’ represent original images and ‘2’ show zoomed‐in, more enhanced versions highlighting changes in cuticle fluorescence.

The hair cut close to the scalp exhibits extremely low fluorescence levels (such images taken at the same exposure conditions can be provided upon request). Blended virgin hair from various hair suppliers often contains pre‐existing weathering and, occasionally, heat damages that are difficult to detect without advanced screening methodologies. Based on the fluorescence images in Figure 3a,b, the virgin hair used in this study already showed typical hair‐weathering markers, resulting in a quite strong baseline fluorescence. Hair exposed to ozonated water for 30 min showed further increased intensity and patchiness, brighter cortical accumulations (likely around melanin/nuclear remnants) in the green channel and emerging granular red in the cortex with cuticle rim intensifying. The green channel image of hair exposed to ozonated water for 360 min contains more very bright green areas in the cortex and some cuticle parts. The red channel image of this hair contains granular cortex hotspots and intense zones in the cuticles. In both channel images, it is possible to observe cuticle areas with eroding fluorescence despite that there is a clear boundary showing physical cuticle limits.

Literature suggests that within filter absorption and emission limits, the data obtained using the green channel could represent (i) oxidized tryptophan → N‐formylkynurenine (NFK) + kynurenine [42] and (ii) advanced lipoxidation end‐products/ALE (advanced lipid peroxidation end‐products that are aldehyde‐protein adducts) [43, 44]. The relationship between ozonated water and tryptophan degradation is already recognized in the literature: the ozone rate constants for reaction for various amino acids, except cysteine, methionine and tryptophan, which react very rapidly, with tryptophan being pointed out by its high reactivities with O_3_, with a k(O_3_)^a^M^−1^s^−1^ value of 7.0 × 10^6^ M^−1^s^−1^, and where the literature points out that tryptophan has the formation of N‐formylkynurenine as the major oxidized product [45, 46, 47].

We could not find studies systematically investigating ALE formation in hair following ozone exposure. However, Valacchi et al. quantified 4‐hydroxy‐2‐nonenal–protein adducts (a type of ALE) in ozonated skin and reported that the relationship between ozone concentration and adduct formation is non‐linear [48]. Another study examined the fluorescence of UV‐oxidized skin resulting from interactions between keratin and oxidized lipids. Mizutani et al. observed increased fluorescence in oxidized stratum corneum within the same GFP‐filtered emission range used in our study [49]. The authors attributed this increase to multiple contributors, including ALE formation, which supports our hypothesis that ALEs are generated both at the hair surface and within the cortex during ozonated‐water exposure [49].

Fluorescence in the red region could indicate the presence of other types of advanced lipoxidation end‐products, which are excited and emitted at higher wavelengths than typical aldehyde‐protein adducts. This interpretation is further supported by studies in wool keratin, an established analogue for human hair, showing that photo‐oxidation of keratin and associated surface lipids generates fluorescent oxidation products with emission extending into the 630–710 nm region [50]. Such red‐shifted fluorophores arise from lipid‐ and cystine‐derived degradation pathways similar to those implicated in ALE formation. Additionally, hair cross‐sections may contain melanin oxidation products that also fluoresce in the red region [51].

### Infrared spectroscopy findings

FTIR spectroscopy is a powerful tool for assessing chemical and structural changes in hair, particularly those induced by oxidative treatments [52]. Figure 4 shows the average FTIR‐ATR spectra of control and ozonated the hair samples. The measurements were conducted on the hair surface, capturing primarily cuticle chemistry and, when cuticles were absent or eroded, limited contributions from the underlying cortex. Because proteins are key structural components governing hair properties, this study focused on several protein‐associated spectral markers.

**FIGURE 4:**
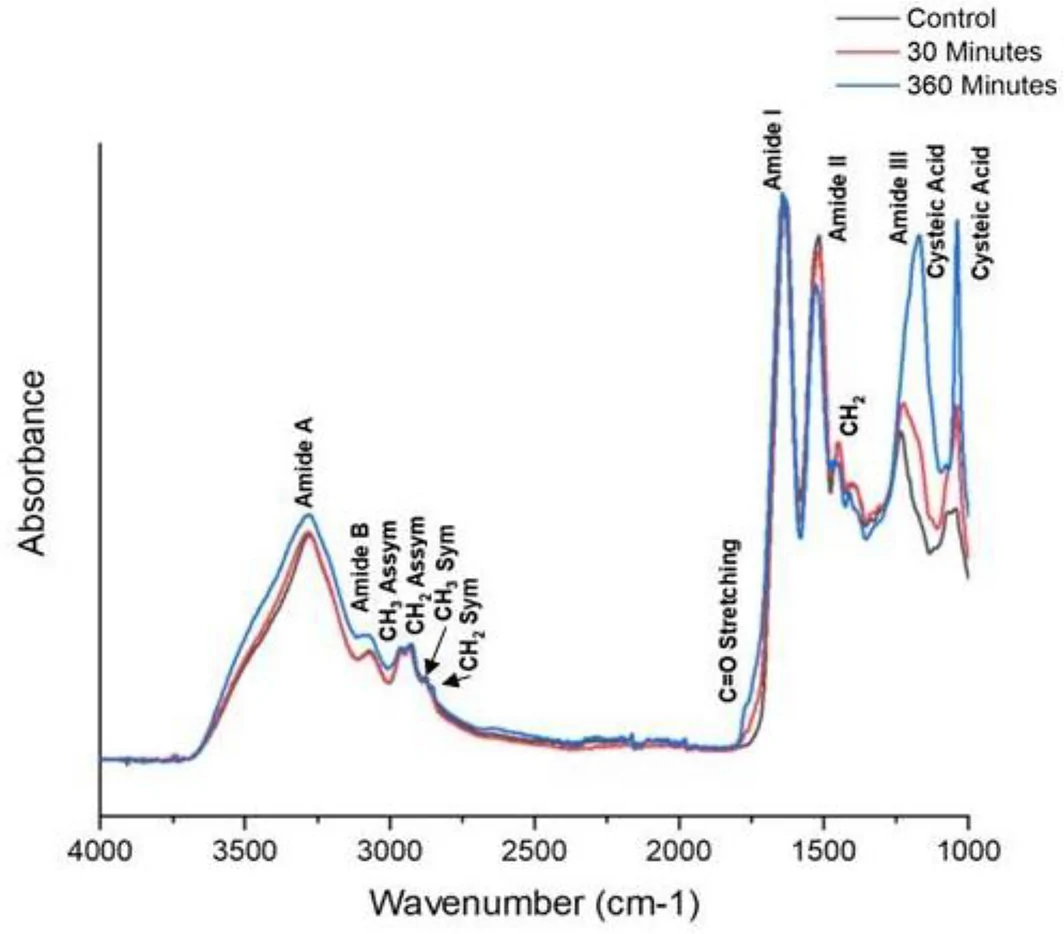
Standard FTIR‐ATR spectra from 1000 to 4000 cm^−1^ comparing samples of control hair and ozonated water washed hair with cycles of 30 and 360 min.

The Amide I region (around 1633 cm^−1^) arises primarily from C=O stretching vibrations, with smaller contributions from C—N, C—C—N and N—H motions. The Amide II region results from N—H bending coupled with C—N stretching [52].

The analysis of the spectra revealed small shifts in the centroids of the bands at 1633 and 1515 cm^−1^ toward higher wavenumbers (Figure 5), indicating subtle alterations in protein secondary structure. In addition, the Amide II band showed a notable reduction in area, suggesting structural degradation of keratin [53], consistent with the observations from SEM imaging and fluorescence microscopy.

**FIGURE 5:**
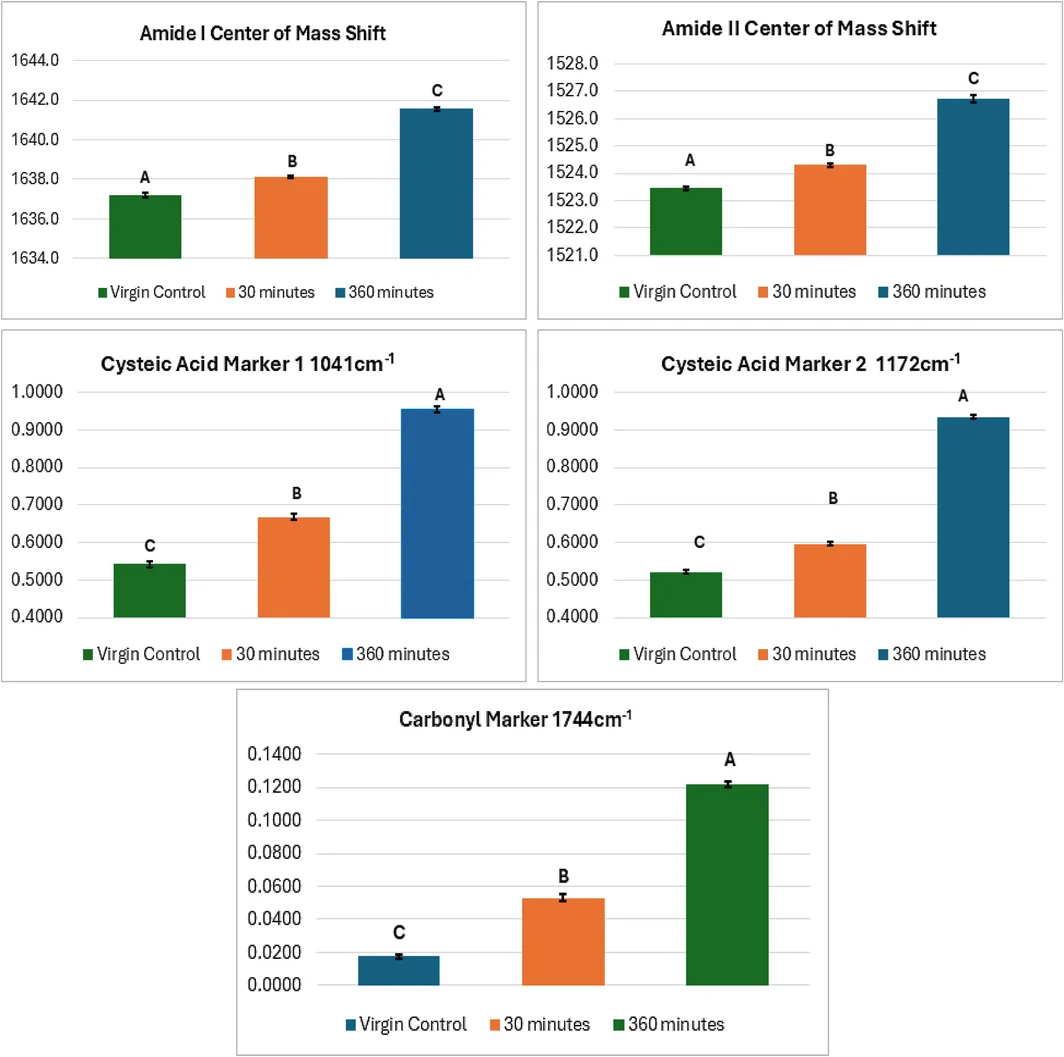
Changes in Amid II and I centre of mass, and the height intensities of the 1041, 1172 and 1744 cm^−1^ peaks. The graph illustrates significant increases and decreases in intensity values, with statistical analysis performed using Tukey's test.

Further changes were identified in regions associated with sulfur‐containing amino acids. Increased intensity at the 1235 cm^−1^ band suggests the formation of oxidative byproducts arising from the interaction between ozone and sulfur‐containing residues. This effect overlapped with the emergence of the 1170 cm^−1^ band, associated with sulfonate groups (S–O asymmetric stretch [54]), particularly cysteic acid—a known oxidation product of cystine (Figure 5). A second cysteic acid band at 1042 cm^−1^ [53, 54] also increased following ozonated‐water exposure. Both features were detectable even after the 30‐min treatment. Similar changes were also observed in our previous study, but with a much smaller impact on the hair fibre when in the dry state and directly exposed to ozone gas, suggesting that the aqueous medium may act as a facilitator of ozone activity [32].

Peak‐height analysis demonstrated increases of 23% and 15% in the 1041 and 1172 cm^−1^ bands, respectively, after 30 min of exposure, and increases of 76% and 80% after 360 min (Figure 5). These concurrent upward trends strongly indicate exposure‐time‐dependent oxidation of sulfur‐containing amino acids at the hair surface. This is consistent with SEM observations and the well‐documented rise in cysteic acid–related peaks following oxidative damage to hair fibres [55, 56] but also corroborates previous findings on ozone‐treated dampened wool fibres, especially concerning the oxidation of cystine residues to sulfonic acid [57].

Special attention should be given to the 1744 cm^−1^ carbonyl band, a marker for aldehydes formed during lipid oxidation [58, 59]. This peak increased by 205% and 603% after 30 and 360 min of ozonated‐water exposure, respectively, with both changes being statistically significant. The substantial rise in carbonyl signal likely reflects aldehydes present in advanced lipid oxidation end‐products (ALEs) accumulated at the hair surface. This aligns with established findings showing that ozone readily induces lipid peroxidation and aldehyde formation [36, 60]. These results support the hypothesis that the increased fluorescence observed in treated fibres may be partly attributable to the formation of fluorescent ALEs during ozone exposure.

### Thermal properties of hair

DSC is a powerful thermoanalytical technique used to investigate structural changes in hair keratin by measuring thermal transitions such as protein denaturation. Two primary approaches are employed in hair analysis: dry DSC, in which the sample is analysed as it undergoes dehydration, and wet DSC, which uses fully hydrated hair fibres. Although dry DSC can provide general information about thermal stability, it often fails to accurately quantify keratin denaturation enthalpy because protein denaturation overlaps with pyrolysis events [61]. By contrast, wet DSC enables more realistic and sensitive detection of keratin denaturation, as the presence of water lowers the denaturation temperature and separates the denaturation and pyrolysis peaks [61].

Within the hair‐science community, denaturation temperature is commonly associated with the density of crosslinking within the fibre, whereas denaturation enthalpy reflects the relative amount of structurally ordered protein [61]. Figure 6 presents the denaturation temperature (Td) and enthalpy values obtained through wet DSC, revealing a significant increase in Td after the first 30‐min ozonated‐water treatment. Ten treatment cycles of 30 min produced an increase of approximately 10C°, suggesting ozonated water may induce chemical changes that enhance thermal stability of hair. A small but statistically significant reduction in denaturation enthalpy was observed after both 30 min and 360 min exposure. Previous research has shown that mild oxidative peroxide damage, after accounting for pH effects, can increase the denaturation temperature of hair [62]. However, with increasing numbers of bleaching cycles, this effect is no longer maintained, whereas hair treated with ozonated water appears to retain enhanced thermal stability even after ten 30‐min cycles.

**FIGURE 6:**
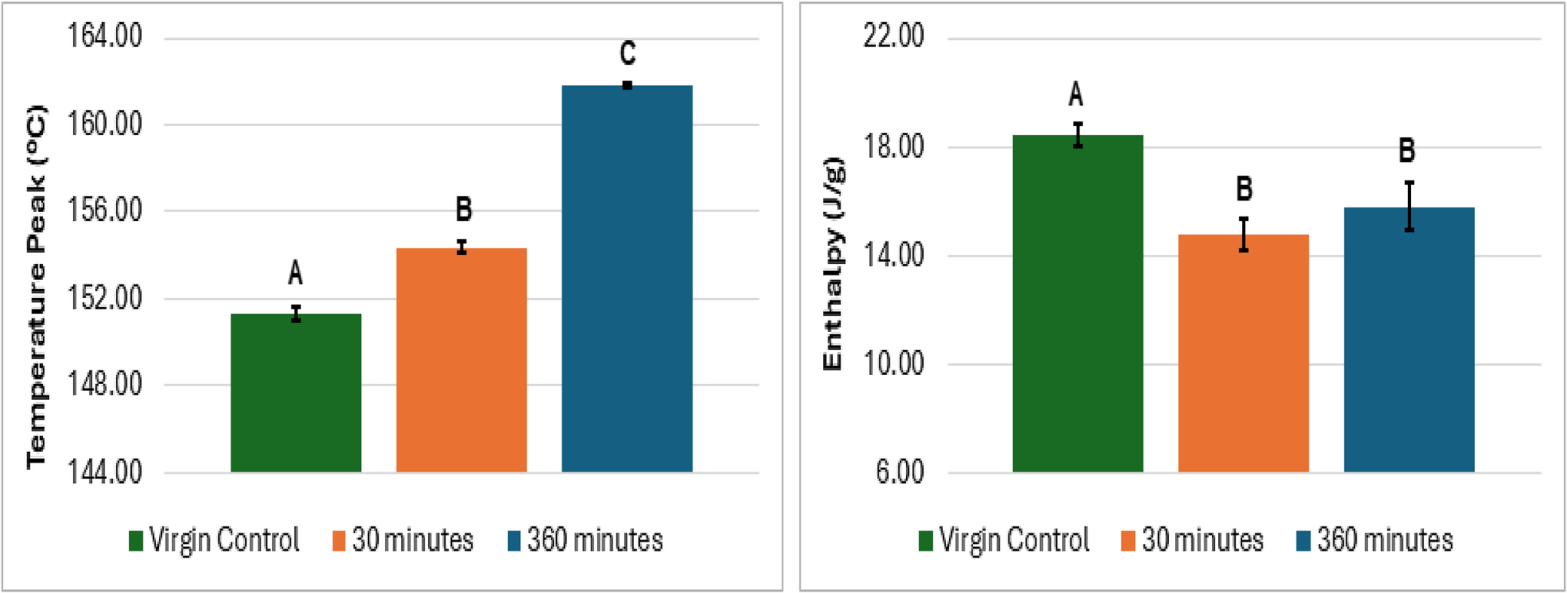
Denaturation temperature and enthalpy of virgin medium brown European hair before and after treatments with ozonated water. Different letters label statistical differences measured using the Tukey HSD test.

The literature suggests that ozone exposure can promote dityrosine formation in proteins [63]. We hypothesize that oxidation of the highly tyrosine‐rich keratin‐associated proteins (KAPs) increases matrix crosslink density, thereby reinforcing the fibre and elevating its thermal stability. Extensive carbonyl formation may also contribute to structural restriction, although mechanistic studies are required to validate these hypotheses.

Meanwhile, denaturation enthalpy exhibited a slight but statistically significant decrease at both treatment times. Although FTIR primarily measures the hair surface, where keratin abundance is relatively low (only keratins acidic keratins K32, K35, K39, K40 and basic keratins K85, K82 are found in the cuticles [64]), the reduction in the Amide II band may reflect meaningful alterations in the secondary structure of these surface keratins. By extrapolation, these FTIR observations align with the decrease in DSC enthalpy. Such reductions in denaturation enthalpy are common following both chemical and physical treatments of hair [54, 65].

### Wet tensile property analysis

Wet tensile property analysis results are presented in Figure 7. Break extension and Young's modulus showed no statistically significant differences among treatment groups. By contrast, both break stress and post‐yield gradient were significantly increased following 360 min of exposure to ozonated water (13% and 36% increases, respectively), indicating enhanced fibre stiffness and strength. These findings support the hypothesis that prolonged ozone exposure promotes additional covalent crosslink formation within the hair matrix, consistent with the observed rise in denaturation temperature.

**FIGURE 7:**
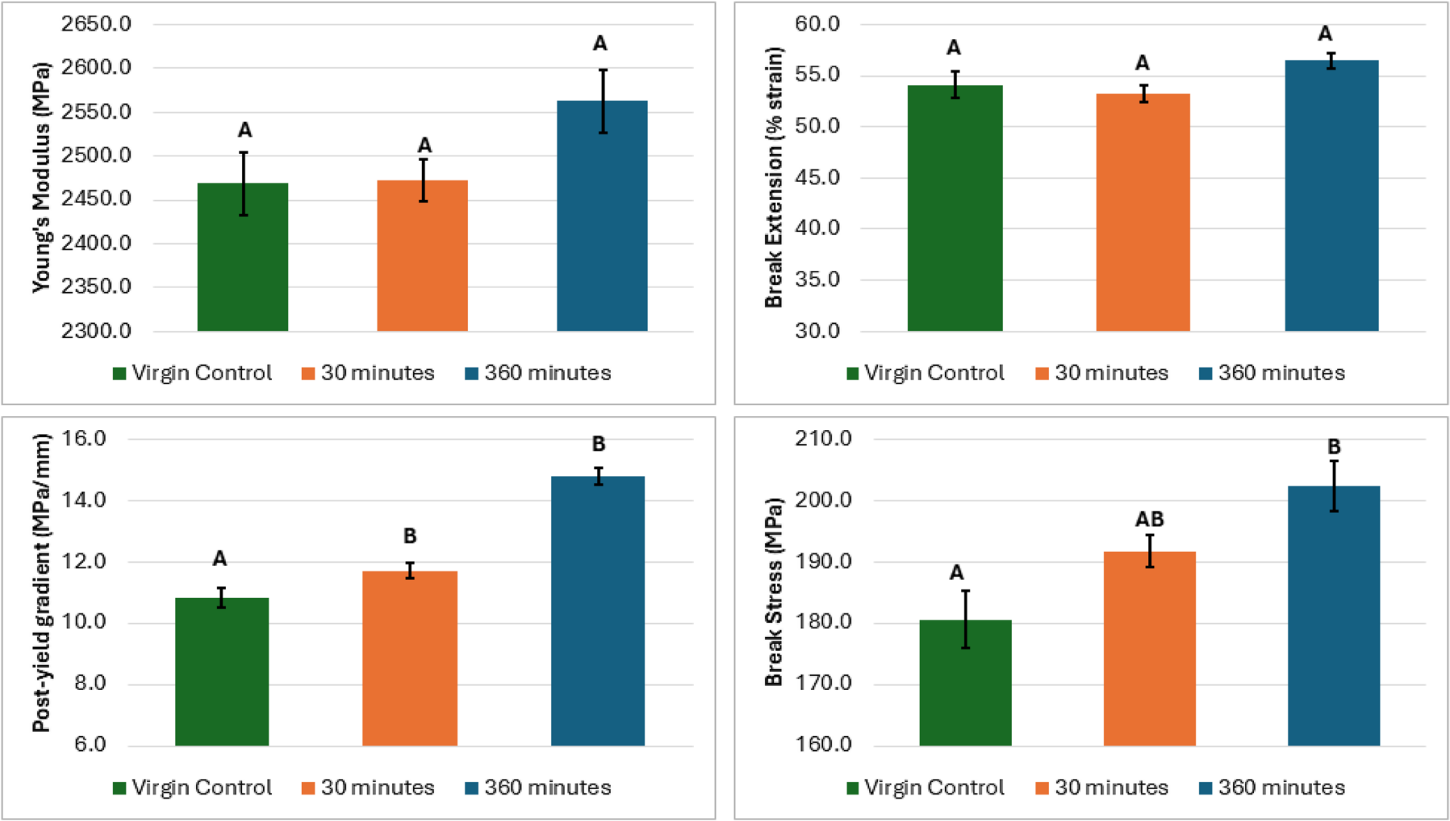
Young modulus, break extension, post‐yield gradient and break stress of differently treated virgin medium brown European hair. Different letters label statistical differences measured using the Tukey HSD test.

Feughelman proposed that the post‐yield slope is an indicator of fibre stiffness, governed by the crosslinks and entanglements between the microfibrils and the surrounding matrix [66]. The post‐yield gradient results obtained in this study are consistent with the denaturation temperature data (correlation coefficient = 0.997) and support the hypothesis that prolonged ozone exposure promotes the formation of additional covalent bonds that reinforce the fibre structure and may contribute to increased tensile strength, especially with the collapse of disulphide bonds and creation of cysteic acids. To our knowledge, this is the first peer‐reviewed report demonstrating post‐yield slope changes in hair following ozonated‐water exposure. However, a poster presented at the Advances in Cosmetic Formulation Design conference reported a similar trend when analysing dry tensile properties of gaseous ozone‐treated hair [67].

It is also noteworthy that, despite exhibiting an effect contrary to what is typically expected from oxidative processes on hair‐fibre strength, ozonated water use did not produce a significant change in the break extension and Young's modulus of the hair fibre. This suggests that, in addition to the potential formation of crosslinks, the effects of ozonated water differ from those induced by other oxidative agents or by heat treatment described in the literature [68].

### Changes in consumer perceived hair attributes

To complement the structural analyses, consumer‐relevant properties, such as shine, colour and dry combing, were evaluated, as these are essential for assessing the cosmetic performance of hair treatments, particularly in the context of marketing claims often associated with ozone exposure. Shine measurements quantify light reflection from the hair surface, which is often promoted as being enhanced by ozone treatments [24, 25, 26, 27]. Colour assessments help determine whether treatments preserve or alter hair colour over time, especially for chemically treated or dyed hair. Dry combing force reflects hair manageability and smoothness, serving as an indicator of hair alignment, surface smoothness and, to some degree, cuticle integrity.

Together, these measurements are critical for substantiating claims such as ‘enhanced shine’, ‘improved colour longevity’ and ‘increased softness/smoothness’, commonly attributed to ozone‐based hair treatments [24, 25, 26, 27]. They also help to bridge the gap between molecular findings and real‐world user experience.

Figure 8 presents the results of these evaluations, comparing the average values before and after hair treatments with ozonated water.

**FIGURE 8:**
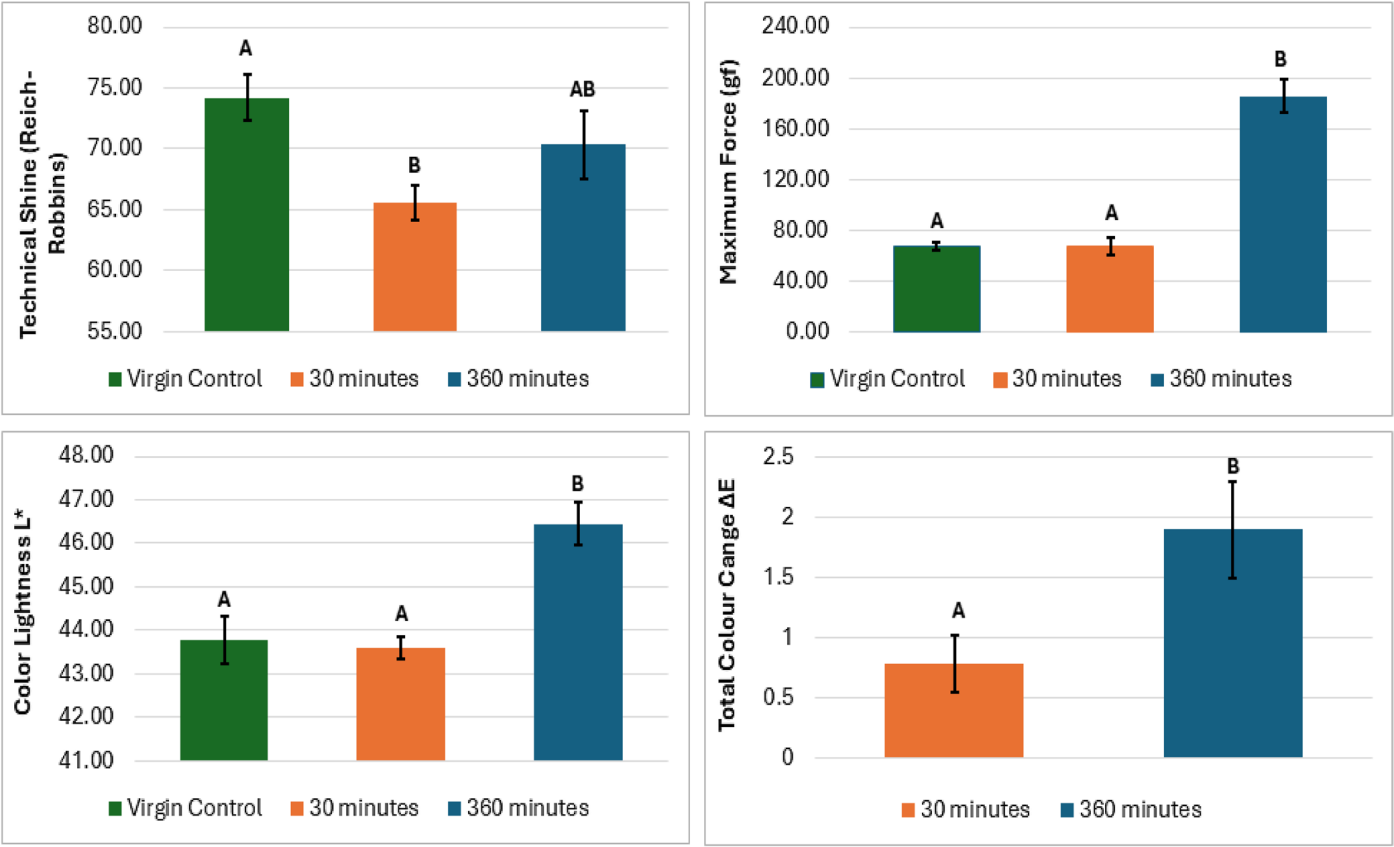
Dry combing, shine and colour analysis of virgin medium brown hair before and after treatment with ozonated water. Different letters label statistical differences measured using the Tukey HSD test.

Measured technical shine showed minor fluctuations in mean values, which are not considered meaningful.

Dry combing force did not show significant changes after 30 min of treatment; however, a 174% increase in combing force was observed after 360 min. This may be associated with cuticle fusion and the substantial alterations in surface chemistry identified in Figures 1 and 6. The negative changes in consumer perceived hair smoothness and manageability have also been reported in studies examining peroxide bleaching of virgin hair [69, 70], and in exaggerated gaseous ozone exposure, which resulted in more than a six‐fold increase in dry combing force [66].

In the CIELAB colour system, L* represents lightness, ranging from 0 (absolute black) to 100 (absolute white) and indicates how light or dark a colour appears [71]. The gradual increase in L* values from the 30‐min time point onward suggests that ozone may have a lightening effect on hair fibres, a phenomenon previously reported in the literature for textiles like wool undergoing ozone‐based bleaching [72], these results also highlight similarities between the two materials. Earlier studies on ozone‐treated wool fabrics, especially in the context of shrink and pilling resistance of knitted textiles, similarly described structural alterations in the cuticular layer. These modifications were primarily attributed to the formation of cysteic acid, the terminal oxidation product of disulfide bonds, along with an increase in the dynamic friction coefficient of the fibres, potentially explaining the observed rise in dry combing force [57, 73]. The colour‐lightening effect observed in this study appears to be substantially weaker than the alkaline peroxide–induced lightening previously demonstrated on virgin hair [74].

Across all analytical approaches, ozonated water produced clear, exposure‐dependent oxidative changes in virgin hair. Even at the earliest stage (30 min), subtle yet measurable alterations were detectable, ranging from increased fluorescence within the cortex to shifts in thermal behaviour, indicating that oxidative effects penetrate beyond the cuticle into deeper fibre regions. These early changes parallel mild oxidative damage reported in the literature and suggest that cumulative exposure can initiate reorganization of both surface and internal keratin structures.

Prolonged exposure (12 times of 30 min cycles) resulted in the most pronounced effects. SEM, optical and fluorescence microscopy revealed extensive cuticle smoothing, fusion and partial loss, accompanied by strong oxidative fluorescence in cortical regions. FTIR confirmed substantial increases in cysteic acid–related peaks and carbonyl content, indicating sulfur‐amino‐acid oxidation and lipid peroxidation. DSC showed elevated denaturation temperature, while mechanical testing revealed higher break stress and post‐yield slope, supporting the formation of additional covalent crosslinks and increased matrix rigidity.

Consumer‐relevant attributes mirrored these structural and chemical findings, with significant increases in dry combing force and mild fibre lightening. Collectively, these results demonstrate that ozonated water induces progressive oxidative modification of proteins and lipids, affecting both surface integrity and internal fibre architecture. The magnitude of these changes underscores the need for further biochemical analyses, such as SDS‐PAGE, HPTLC or lipidomic profiling, to confirm the molecular pathways implicated and to more fully align these observations with known ozone‐induced reactions in keratin‐based fibres.

## CONCLUSION

This study demonstrates that repeated exposure to ozonated water induces both superficial and internal modifications within the hair fibre, producing oxidative changes in the cuticle and cortex. A single 30‐min treatment resulted in only subtle alterations; however, repeated exposure (12 × 30‐min cycles) caused pronounced structural and chemical changes, including (i) cuticle fusion visible by SEM, accompanied by a more than two‐fold increase in hair friction; (ii) enhanced cortical fluorescence consistent with tryptophan degradation and the formation of advanced lipid peroxidation products, supported by significant increases in carbonyl content detected via FTIR; (iii) elevated keratin denaturation temperature together with increased post‐yield stiffness and break stress, suggesting the formation of additional covalent crosslinks within the hair matrix; and (iv) measurable fibre lightening, indicating partial melanin degradation. While this work provides new insight into the relationship between ozone‐induced structural changes and alterations in hair protein and pigment chemistry, further research is needed to characterize how ozonated water affects individual lipid classes. A clearer understanding of lipid‐specific oxidation pathways will also help predict changes in scalp and fibre lipid composition following ozone exposure.

From a practical perspective, many of the altered properties identified here are not perceptible to consumers. Instead, individuals undergoing repeated ozonated water treatments are likely to experience increased roughness and tangling, together with some colour lightening. These findings underscore the importance for hairdressing salons to recognize that repeated ozonated water use may lead to gradual and largely irreversible deterioration of virgin hair. Future work should investigate how previously damaged hair responds to such treatments, how ozone‐modified fibres endure subsequent chemical and mechanical stressors and how cosmetic formulations interact with hair that has undergone ozone‐induced structural modification.

## CONFLICT OF INTEREST STATEMENT

No potential conflict of interest was reported by the authors.

## Data Availability

The data that support the findings of this study are available from the corresponding author upon reasonable request.

## References

[1] Stenn K . Hair: a human history. New York: Pegasus Books; 2016.

[2] Lima CRRC , Couto RAA , Freire TB , Goshiyama AM , Baby AR , Velasco MVR , et al. Heat‐damaged evaluation of virgin hair. Journal of Cosmetic Dermatology. 2019;18(6):1885–1892. 10.1111/jocd.12892 30861299

[3] Velasco MVR , Dias TCS , Freitas AZ , Júnior NDV , Pinto CASO , Kaneko TM , et al. Hair fiber characteristics and methods to evaluate hair physical and mechanical properties. Brazilian Journal of Pharmaceutical Sciences. 2009;45:153–162.

[4] Mauricio LPA . Caracterização da integridade estrutural da fibra capilar tratada com diferentes produtos químicos [Dissertação de mestrado, Universidade Federal do Rio Grande do Norte]. 2019.

[5] Bhushan B . Biophysics of human hair. Berlin, Heidelberg: Springer; 2010.

[6] Robbins CR . Chemical aspects of bleaching human hair. Journal of the Society of Cosmetic Chemists. 1971;22:339–348.

[7] Kaplin IJ , Schwan A , Zahn H . Effects of cosmetic treatments on the ultrastructure of hair. Cosmet Toiletries. 1982;97(8):22–25.

[8] Wagner RDC , Joekes I . Hair medulla morphology and mechanical properties. Journal of Cosmetic Science. 2007;58(4):359–368. 17728936

[9] Trüeb RM . The impact of oxidative stress on hair. International Journal of Cosmetic Science. 2015;37(S2):25–30. 10.1111/ics.12286 26574302

[10] Leon BR , Romary DJ , Landsberger SA , Bradner KN , Ramirez M , Lubitz RM . Risks of ozonated oil and ozonated water on human skin: a systematic review. International Wound Journal. 2022;19(7):1901–1910. 10.1111/iwj.13760 PMC9615280 35083865

[11] Breidablik HJ , Lysebo DE , Johannessen L , Skare Å , Andersen JR , Kleiven OT . Ozonized water as an alternative to alcohol‐based hand disinfection. Journal of Hospital Infection. 2019;102(4):419–424. 10.1016/j.jhin.2019.01.026 30731184

[12] Restaino L , Frampton EW , Hemphill JB , Palnikar P . Efficacy of ozonated water against various food‐related microorganisms. Applied and Environmental Microbiology. 1995;61(9):3471–3475. 10.1128/aem.61.9.3471-3475.1995 PMC167626 7574656

[13] Xue W , Macleod J , Blaxland J . The use of ozone technology to control microorganism growth, enhance food safety and extend shelf life: a promising food decontamination technology. Foods. 2023;12(4):814. 10.3390/foods12040814 PMC9957223 36832889

[14] Pandiselvam R , Kaavya R , Jayanath Y , Veenuttranon K , Lueprasitsakul P , Divya V , et al. Ozone as a novel emerging technology for the dissipation of pesticide residues in foods—a review. Trends in Food Science and Technology. 2022;97:38–54.

[15] Romary DJ , Landsberger SA , Bradner KN , Ramirez M , Leon BR . Liquid ozone therapies for the treatment of epithelial wounds: a systematic review and meta‐analysis. International Wound Journal. 2023;20(4):1235–1252. 10.1111/iwj.13941 PMC10031250 36056800

[16] Epelle EI , Macfarlane A , Cusack M , Burns A , Okolie JA , Mackay W , et al. Ozone application in different industries: a review of recent developments. Chemical Engineering Journal. 2023;454:140188. 10.1016/j.cej.2022.140188 PMC9637394 36373160

[17] Ding W , Jin W , Cao S , Zhou X , Wang C , Jiang Q , et al. Ozone disinfection of chlorine‐resistant bacteria in drinking water. Water Research. 2019;160:339–349. 10.1016/j.watres.2019.05.014 31158616

[18] Rangel K , Cabral FO , Lechuga GC , Carvalho JPRS , Villas‐Bôas MHS , Midlej V , et al. Detrimental effect of ozone on pathogenic bacteria. Microorganisms. 2022;10(1):40. 10.3390/microorganisms10010040 PMC8779011 35056489

[19] Silva EJNL , Prado MC , Soares DN , Hecksher F , Martins JNR , Fidalgo TKS . The effect of ozone therapy in root canal disinfection: a systematic review. International Endodontic Journal. 2020;53:317–332. 10.1111/iej.13229 31587303

[20] Pires MV , de Lima CJ , Carvalho HC , Moreira LH , Fernandes AB . Effectiveness of intravesical ozone in interstitial cystitis by the O'Leary–Sant symptom index. International Urogynecology Journal. 2023;34:1437–1446. 10.1007/s00192-022-05383-3 PMC9569001 36242631

[21] Zeng J , Lu J . Mechanisms of action involved in ozone‐therapy in skin diseases. International Immunopharmacology. 2018;56:235–241. 10.1016/j.intimp.2018.01.040 29414657

[22] Ozbay I , Ital I , Kucur C , Akcılar R , Deger A , Aktas S , et al. Effects of ozone therapy on facial nerve regeneration. Brazilian Journal of Otorhinolaryngology. 2017;83(2):168–175. 10.1016/j.bjorl.2016.02.009 PMC9442687 27174776

[23] Shah P , Shyam AK , Shah S . Adjuvant combined ozone therapy for extensive wound over tibia. Indian Journal of Orthopaedics. 2011;45:376–379. 10.4103/0019-5413.80332 PMC3134027 21772635

[24] Instagram . #ozonehair [Hashtag page]. 2025 [cited 2025 Aug 21]. Available from: https://www.instagram.com/explore/tags/ozonehair/

[25] The benefits of ozone hair treatments [Internet]. Zahra Salon & Spa. 2023 [cited 2025 Aug 21]. Available from: https://zahrasalonandspa.ca/the‐benefits‐of‐ozone‐hair‐treatments/

[26] What is ozone therapy? How it works — explore the benefits of the steam‐based hair treatment [Internet]. Revista Marie Claire. 2022 [cited 2025 Aug 21]. Available from: https://revistamarieclaire.globo.com/Beleza/Cabelo/noticia/2022/01/o‐que‐e‐ozonioterapia‐como‐funciona‐conheca‐os‐beneficios‐do‐tratamento‐capilar‐feito‐vapor.html

[27] Ozone therapy treatment hair [Internet]. TikTok. 2025 [cited 2025 Aug 21]. Available from: https://www.tiktok.com/discover/ozone‐therapy‐treatment‐hair

[28] Cirlini M , Caligiani A , Palla G , de Ascentiis A , Tortini P . Stability studies of ozonized sunflower oil and enriched cosmetics with a dedicated peroxide value determination. Ozone: Science & Engineering. 2012;34(4):293–299.

[29] Radzimierska‐Kaźmierczak M , Śmigielski K , Sikora M , Nowak A , Plucińska A , Kunicka‐Styczyńska A , et al. Olive oil with ozone‐modified properties and its application. Molecules. 2021;26(11):3074. 10.3390/molecules26113074 PMC8196576 34063952

[30] Hanada K , Okuda D , Ogi R , Kojima S , Tsuruoka R , Shiota G . Ozonized glycerin (OG)‐based cosmetic products lighten age spots on human facial skin. Journal of Cosmetic Dermatology. 2022;21(7):3133–3139. 10.1111/jocd.15002 PMC9544616 35435300

[31] Peng Y , Chen Z , Li Y , Wang Y , Ye C , Xu J , et al. Bacteriostasis and cleaning effect of trace ozone replacing personal care products. Environmental Technology. 2022;44(17):1–14. 10.1080/09593330.2022.2036818 35099355

[32] Laurindo Igreja Marrafa PA , Kawata BA , Braga Carneiro H , de Souza Botelho T , Carvalho HC , de Castro Lima CRR , et al. Evaluation of the ozone effects on human hair fiber: a preliminary in vitro study. Ozone: Science & Engineering. 2024;46(3):230–241.

[33] Murphy L . Ozone—the latest advance in sterilization of medical devices. Canadian Operating Room Nursing Journal. 2006;24(2):28–38. 16869464

[34] Ferrara F , Pambianchi E , Woodby B , Messano N’ , Therrien JP , Pecorelli A , et al. Evaluating the effect of ozone in UV induced skin damage. Toxicology Letters. 2021;338:40–50. 10.1016/j.toxlet.2020.11.023 33279629

[35] Weschler CJ , Nazaroff WW . Human skin oil: a major ozone reactant indoors. Environmental Science: Atmospheres. 2023;3(4):640–661.

[36] Pandrangi LS , Morrison GC . Ozone interactions with human hair: ozone uptake rates and product formation. Atmospheric Environment. 2008;42(20):5079–5089.

[37] Thiele JJ , Podda M , Packer L . Tropospheric ozone: an emerging environmental stress to skin. Biological Chemistry. 1997;378(11):1299–1305. 10.1515/bchm.1997.378.11.1299 9426190

[38] Van Leeuwen J . Proposed OS&E Requirement: measuring ozone dosage. Ozone: Science & Engineering. 2015;37(2):191–192. 10.1080/01919512.2015.1006467

[39] Robinson VNE . A study of damaged hair. Journal of the Society of Cosmetic Chemists. 1976;27:155–161.

[40] Yun JH , Kim HJ , Lee YJ , Park CC . Physical properties of human hair by the bleach. Journal of the Korean Society of Clothing and Textiles. 2005;7(1):96–100.

[41] Robbins CR . Bleaching and oxidation of human hair. Chemical and Physical Behavior of Human Hair. Berlin, Heidelberg: Springer Berlin Heidelberg; 2012. p. 282.

[42] Jachowicz J , McMullen RL . Tryptophan fluorescence in hair—examination of contributing factors. Journal of Cosmetic Science. 2011;62:291–304. 21839032

[43] Li W , Gao H , Mu H , Chen H , Fang X , Zhou Y , et al. Three different active aldehydes induce the production of advanced lipoxidation end products upon incubation with bovine serum albumin. European Journal of Lipid Science and Technology. 2015;117(9):1432–1443.

[44] Lamore SD , Azimian S , Horn D , Anglin BL , Uchida K , Cabello CM , et al. The malondialdehyde‐derived fluorophore DHP‐lysine is a potent sensitizer of UVA‐induced photooxidative stress in human skin cells. Journal of Photochemistry and Photobiology B: Biology. 2010;101(3):251–264. 10.1016/j.jphotobiol.2010.07.010 PMC2963670 20724175

[45] Sharma VK , Graham NJD . Oxidation of amino acids, peptides, and proteins by ozone: a review. Ozone: Science & Engineering. 2010;32(2):81–90. 10.1080/01919510903510507

[46] Kanofsky JR , Sima P . Singlet oxygen production from the reactions of ozone with biological molecules. The Journal of Biological Chemistry. 1991;266(14):9039–9042. 2026612

[47] Cataldo F . On the action of ozone on proteins. Polymer Degradation and Stability. 2003;82(1):105–114. 10.1016/S0141-3910(03)00170-8

[48] Valacchi G , Fortino V , Bocci V . The dual action of ozone on the skin. British Journal of Dermatology. 2005;153(6):1096–1100. 10.1111/j.1365-2133.2005.06939.x 16307642

[49] Mizutani T , Sumida H , Sagawa Y , Okano Y , Masaki H . Carbonylated proteins exposed to UVA and to blue light generate reactive oxygen species through a type I photosensitizing reaction. Journal of Dermatological Science. 2016;84(3):314–321. 10.1016/j.jdermsci.2016.09.016 27743910

[50] Collins S . Studies on the natural fluorescence of wool and wool grease. 1992. Doctoral thesis, City University London. Available from: https://openaccess.city.ac.uk/id/eprint/7894/

[51] Nighswander‐Rempel SP , Riesz J , Gilmore J , Bothma JP , Meredith P . Quantitative fluorescence excitation spectra of synthetic eumelanin. The Journal of Physical Chemistry. B. 2005;109(43):20629–20635. 10.1021/jp053704+ 16853670

[52] Zhang G , McMullen RL , Mendelsohn R , Musa O . Applications of vibrational spectroscopic imaging in personal care studies. New York: CRC Press; 2015. p. 1–26.

[53] Foggia MD , Boga C , Micheletti G , Nocentini B , Taddei P . Vibrational Raman and IR data on brown hair subjected to bleaching. Data in Brief. 2021;38:107439. 10.1016/j.dib.2021.107439 PMC8502914 34660859

[54] Gillece T , Senak L , RL MM . Characterization of bleached hair: vibrational spectroscopy, thermal analysis, and determination of equivalent damage factor. Journal of Cosmetic Science. 2021;72:519–546.

[55] Enami S , Hoffmann MR , Colussi AJ . Simultaneous detection of cysteine sulfenate, sulfinate, and sulfonate during cysteine interfacial ozonolysis. The Journal of Physical Chemistry. B. 2009;113(28):9356–9358. 10.1021/jp904316n 19537744

[56] Signori V , Lewis DM . FTIR investigation of the damage produced on human hair by weathering and bleaching processes: implementation of different sampling techniques and data processing. International Journal of Cosmetic Science. 1997;19(1):1–13. 10.1046/j.1467-2494.1997.171752.x 18507645

[57] Thorsen WJ . Ozone wool shrinkproofing systems. Ozone Science & Engineering. 1979;1(3):219–233.

[58] Bujok J , Gąsior‐Głogowska M , Marszałek M , Trochanowska‐Pauk N , Zigo F , Pavľak A , et al. Applicability of FTIR‐ATR method to measure carbonyls in blood plasma after physical and mental stress. BioMed Research International. 2019;2019(1):2181370. 10.1155/2019/2181370 PMC6457301 31032337

[59] Oleszko A , Olsztyńska‐Janus S , Walski T , Grzeszczuk‐Kuć K , Bujok J , Gałecka K , et al. Application of FTIR‐ATR spectroscopy to determine the extent of lipid peroxidation in plasma during haemodialysis. BioMed Research International. 2015;2015(1):245607. 10.1155/2015/245607 PMC4417580 25961007

[60] Pryor WA . Mechanisms of radical formation from reactions of ozone with target molecules in the lung. Free Radical Biology and Medicine. 1994;17(5):451–465. 10.1016/0891-5849(94)90172-4 7835752

[61] Wortmann FJ , Springob C , Sendelbach G . Investigations of cosmetically treated human hair by differential scanning calorimetry in water. Journal of Cosmetic Science. 2002;53(4):219–228. 12219248

[62] Marsh JM , Clarke CJ , Meinert K , Dahlgren RM . High‐pressure differential scanning calorimetry of colorant products. Journal of Cosmetic Science. 2007;58(6):621–627. 18305875

[63] Kampf CJ , Liu F , Reinmuth‐Selzle K , Berkemeier T , Meusel H , Shiraiwa M , et al. Protein cross‐linking and oligomerization through dityrosine formation upon exposure to ozone. Environmental Science & Technology. 2015;49(18):10859–10866. 10.1021/acs.est.5b02902 26287571

[64] Plowman JE . Diversity of trichocyte keratins and keratin associated proteins. The Hair Fibre: Proteins, Structure and Development. Singapore: Springer Singapore; 2018. p. 21–32. 10.1007/978-981-10-8195-8_3 29797265

[65] Mcmullen RL , Zhang G , Gillece T . Quantifying hair shape and hair damage induced during reshaping of hair. Journal of Cosmetic Science. 2015;66(6):379–409. 27125013

[66] Feughelman M . The post‐yield region and the structure of keratin. Textile Research Journal. 1964;34(6):539–545.

[67] Malinauskyte E , Wyluda K , Mac B , Gourion‐Arsiquaud S . Characterization of ozone effect on human hair. 2018 Poster. Advances in Cosmetic Formulation Design.

[68] Wortmann FJ , Quadflieg JM , Wortmann G . Comparing hair tensile testing in the wet and the dry state: possibilities and limitations for detecting changes of hair properties due to chemical and physical treatments. International Journal of Cosmetic Science. 2022;44(4):421–430. 10.1111/ics.12796 PMC9544838 35689553

[69] Garcia ML , Diaz J . Combability measurements on human hair. Journal of the Society of Cosmetic Chemists. 1976;27(9):379–398.

[70] Weiand E , Rodriguez‐Ropero F , Roiter Y , Angioletti‐Uberti S , Dini D , Ewen JP . Understanding and controlling the friction of human hair. Advances in Colloid and Interface Science. 2025;345:103580. 10.1016/j.cis.2025.103580 40782659

[71] Vaughn M , van Oorschot R , Baindur‐Hudson S . Hair color measurement and variation. American Journal of Physical Anthropology. 2008;137(1):91–96. 10.1002/ajpa.20849 18446854

[72] Gülümser T , Akça C , Bahtiyari Mİ . Investigation of the effects of ozone on whiteness degree in wool finishing. Textile and Apparel. 2009;19(1):52–55.

[73] Wan A , Yu W . Effect of wool fiber modified by ecologically acceptable ozone‐assisted treatment on the pilling of knit fabrics. Textile Research Journal. 2012;82(1):27–36.

[74] De Morales IR , Breakspear S , Neu A , Bauer P , Lipinski C , Noecker B . Internal damage analysis of Caucasian hair induced by bleaching treatments: a comprehensive comparison via different analytical techniques. Journal of Cosmetic Science. 2024;75(4):243–262.

